# The key technologies of a computer-aided design system for removable partial denture frameworks

**DOI:** 10.1371/journal.pone.0340057

**Published:** 2026-02-03

**Authors:** Guidian Ma, Xiaotong Jiang, Ning Dai, Zhilei Wu, Song Ying, Zongjun Tian

**Affiliations:** 1 College of Mechanical and Electrical Engineering, Nanjing University of Aeronautics and Astronautics, Nanjing, Jiangsu, China; 2 College of Mechanical Engineering, Changshu Institute of Technology, Changshu, Jiangsu, China; Universiti Sains Malaysia, MALAYSIA

## Abstract

This paper presents a computer-aided design (CAD) system for removable partial denture (RPD) frameworks, addressing the challenges of dentition defects. The system takes a digitized dental model obtained via optical scanning as input and generates an RPD framework model ready for 3D printing. Key technologies include spline curve editing and modeling, mesh offsetting, texture image-based modeling, and component models fusion. The system utilizes conformal mapping between the dental model and a disk. This enables spline curve editing to be executed in the parameterized 2D domain, ensuring both accuracy and efficiency. An iterative approximation method with adaptive mesh simplification is introduced to achieve precise mesh offsetting while avoiding self-intersections. Furthermore, texture mapping enables interactive modeling of holes for denture base connectors and 3D branch-like wax patterns for major connectors. An enhanced Boolean algorithm, combined with smoothing and simplifying techniques for intersecting regions, is utilized to ensure seamless and natural integration of various components. Clinical evaluations demonstrate that the system achieves a performance level comparable to advanced commercial CAD systems, having successfully completed over 30,000 clinical designs with high reliability and meeting all required standards.

## 1. Introduction

Products in the field of dentistry are highly personalized. With the widespread adoption of intraoral scanning technology, the digitalization of dentistry has progressed rapidly. Methods such as computer-aided design (CAD) and finite element analysis (FEA) have been extensively studied and applied, with CAD technology in particular achieving significant advancements in the dental domain. [1–9]. Among the primary approaches to addressing dentition defects is the fabrication of removable partial denture (RPD) frameworks [10]. In recent years, driven by the continuous advancement of material technologies and the increasing demands of aging societies in many countries, the digital design of RPD frameworks has undergone rapid development. This progress has yielded significant social and economic benefits, highlighting the growing importance of CAD in modern prosthodontics [11].

RPD framework is a prosthesis designed to restore partial tooth loss, which can be easily removed and reattached by the patient. It comprises several key components, including rests, retainers, connectors, bases, and artificial teeth, which collectively restore the integrity of the dental arch and chewing function. Denmark’s 3Shape company was the first to introduce specialized software for the digital design of RPD frameworks. This software streamlines the design process through a structured procedural workflow, allowing technicians to acquire basic framework design skills with minimal training. However, the core technologies underpinning the software remain undisclosed due to commercial confidentiality.

The design of RPD frameworks primarily involves drawing spline curves on dental models to construct various components. This process integrates key operations such as mesh surface offsetting, texture mapping, and component model fusion to achieve the final framework design. The effectiveness of these technologies significantly influences the quality and efficiency of RPD framework design. Consequently, the development of high-quality algorithms is critical to advancing CAD systems for RPD frameworks.

Based on discrete mesh digital geometry processing technology, this paper presents a computer-aided design (CAD) system for RPD frameworks. The main contributions of this work are summarized as follows:

(1) Comprehensive CAD System for RPD Frameworks

A commercial CAD system for RPD framework design is proposed, incorporating advanced digital geometry processing technologies. The system supports the design of various components, including denture base connectors, minor connectors, major connectors, lingual bars, rests, clasps, finish lines, wax patterns, and cast stops. Key technologies utilized include spline curve editing and modeling, mesh surface offsetting, texture image mapping, and Boolean operations. The developed software has already been successfully applied in clinical practice.

(2) Spline Curve Editing and Modeling

Spline curve editing and modeling are central to the software, significantly influencing user experience. This paper introduces a spline curve editing and modeling method based on conformal mapping parameterization. The method employs a discrete conformal mapping technique to parameterize the 3D dental model onto a planar disk. Control points selected from the 3D model are mapped onto the parameterized plane, where spline curves are calculated and subsequently projected back onto the 3D surface. This approach facilitates the generation of high-quality splines on 3D surfaces and supports real-time surface spline editing. Additionally, a spline modeling technique constrained by 3D mesh surfaces is introduced, enabling real-time modeling of components such as minor connectors, rests, and clasps.

(3) High-Quality Mesh Surface Offsetting

To ensure precise mesh surface offsetting, an iterative approximation method is employed, which maintains offset accuracy while preventing self-intersections. During the iterative process, adaptive mesh simplification is applied to enhance stability and computational efficiency.

(4) Texture Image Mapping for Denture Base Connectors

A texture image mapping method based on As-Rigid-As-Possible (ARAP) parameterization is employed, allowing for real-time editing of mesh positions on denture base connectors. By combining local mesh subdivision with deformation techniques, this method enables the generation of 3D branch-like wax patterns structures on major connectors.

(5) Component Integration Using Boolean Operations

Efficient and robust Boolean operations are implemented to integrate various framework components seamlessly. Smoothing and simplification of intersecting regions are performed to ensure high-quality merged areas, enhancing the overall structural integrity and aesthetic quality of the design.

## 2. System overview

**Fig 1:**
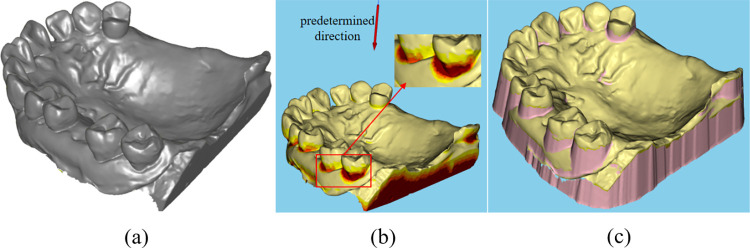
Dental scan data and repaired data. **(a)** Preliminary repaired dental model. **(b)** The undercut area by a predetermined direction. **(c)** The new model without undercut area.

The input to the CAD system for RPD frameworks is a single dental model, typically obtained using an optical scanner. This model is commonly represented as a triangular mesh and stored in file formats such as STL or PLY. Fig 1(a) shows the dental model after preliminary repair. The repaired model must meet specific criteria, ensuring the absence of non-manifold elements, holes, and topological defects. Once these conditions are satisfied, a removal undercut operation, defined by a specified angle and a predetermined direction, is applied to generate a new model, as shown in Fig 1(b) and Fig 1(c). Fig(b) shows the undercut area on the model when a predetermined direction is defined. Fig 1(c) shows the new model which is homeomorphic to a circle, and the subsequent design process proceeds on this model.

**Fig 2:**
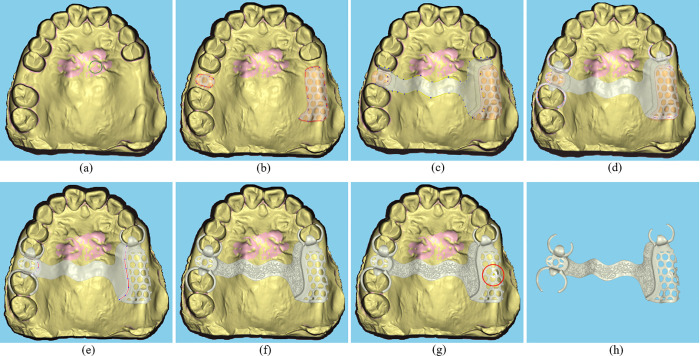
The design process of a removable partial denture (RPD) framework. **(a)** Local model repair. **(b)** Denture base connectors generation. **(c)** Major connectors generation. **(d)** Minor connectors, clasps, etc., generation. **(e)** Finish lines generation. **(f)** 3D branch-like wax patterns. **(g)** Cast stop addition. **(h)** Framework model.

The RPD framework design process is outlined in Fig 2 and consists of the following five primary steps:

(1) Denture Base Connector Generation

The denture base connector area is defined on the dental model using spline curves. Mesh offsetting techniques generate the denture base connectors and underlying wax layer, while texture image mapping defines the positions of the holes. Cylindrical structures are created, and Boolean operations complete the hole-drilling process, as shown in Fig 2(b).

(2) Major Connectors and others Modeling

Closed spline curves are used to extract the areas corresponding to the major connector, rests, and other components. These components are modeled using a mesh non-uniform offsetting technique, as shown in Fig 2(c).

(3) Minor connectors and others Modeling

Open spline curves are employed for real-time modeling and editing of the lingual bar, minor connectors, clasps, finish lines, and other components. Examples of these processes are shown in Fig 2(d) and 2(e).

(4) Component Integration

Boolean operations are applied to seamlessly merge the component models, including the denture base connectors, major and minor connectors, lingual bar, clasps, and finish lines. This integration ensures structural consistency, as illustrated in Fig 2(c), 2(d), and 2(e).

(5) Surface Texture Modeling

Texture image mapping, combined with local mesh subdivision techniques, generates 3D branch-like patterns on the surface of the framework. This enhances both its functionality and aesthetics, as shown in Fig 2(f).

To further enhance usability and efficiency, the software system integrates several auxiliary functions, such as localized model repair (Fig 2(a)), interactive cast stop addition (Fig 2(g)), thickness measurement, and collision detection. Building on these functionalities, this paper highlights four key technologies essential for the design of removable partial denture (RPD) frameworks: spline curve editing and modeling, mesh offsetting, texture mapping-based modeling, and component model fusion.

## 3. Spline curve editing and modeling

In the RPD framework design software, spline curves constitute the foundation for nearly all component modeling processes. Their primary function is to delineate the position and boundaries of each component on the dental model, ensuring precise and efficient design. Based on the modeling principles of component generation, surface spline curves fulfill two critical roles:

Closed Spline Curves: These are employed to segment the dental model into distinct regions. Mesh offsetting techniques are subsequently applied to create components such as denture base connectors and major connectors, ensuring structural integrity and design accuracy.

Open Spline Curves: These are utilized for dynamic construction of component models along the spline direction on the dental model. Typical examples include lingual bars, minor connectors, clasps, and finish lines, which require precise control and adaptability during the modeling process.

To enhance usability and streamline the design workflow, the software system integrates real-time spline curve editing and modeling capabilities. These functionalities are crucial for providing an efficient and user-friendly design experience. Consequently, the development of high-performance and robust spline editing algorithms is pivotal to the system’s overall effectiveness.

### 3.1. Spline curve editing

Spline curves are fundamental in geometric modeling [12,13], serving crucial roles in the design, interaction, and editing of freeform surfaces. They are extensively applied in advanced geometric operations such as surface intersection [14], trimming [15], and CNC toolpath planning for machined surfaces [16,17]. On mesh surfaces, curves are composed of discrete line segments constrained to the surface, making their computation challenging. Existing methods for curve design on mesh surfaces can be broadly categorized into three main approaches: projection methods [18,19], smoothing methods [20,21], and parameterization methods [22,23].

Projection Methods: Curves are initially designed in Euclidean space and then iteratively mapped onto the mesh surface. While straightforward to implement, these methods are computationally expensive and prone to producing multiple solutions on complex surfaces, reducing robustness.

Smoothing Methods: Smoothness constraints are relaxed, and manifold-constrained, energy-driven optimization is applied to refine initial curves. This approach generates smoother curves but requires multiple iterations, limiting computational efficiency and real-time interactivity.

Parameterization Methods: Curves are designed in the planar parameter domain of a 3D model and subsequently mapped back onto the mesh surface. By working in the 2D domain, these methods enable efficient and stable curve editing, making them particularly suitable for complex models.

In this study, we adopt the parameterization method for spline curve editing on dental models. To minimize distortion during the mapping between 2D and 3D domains, a discrete conformal mapping technique [24] is employed. This method maps the boundary of the dental model onto a circle, allowing 3D spline curve design to be conducted within a planar disk.

**Fig 3:**
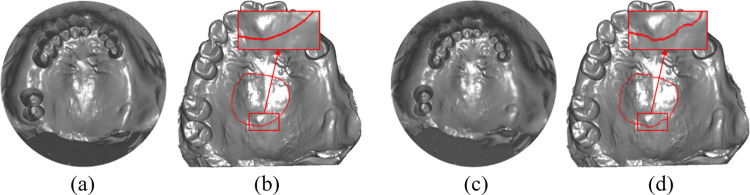
Parameterization results using fixed boundary method. **(a)** Discrete conformal mapping parameterization result. **(b)** Spline curve design based on discrete conformal mapping parameterization. **(c)** Centroid mapping parameterization result. **(d)** Spline curve design based on centroid mapping parameterization.


Fig 3(a) and 3(c) illustrate the results of discrete conformal mapping and centroid mapping [25] of the dental model, respectively. Fig 3(b) and 3(d) display the corresponding spline curve design outcomes. The results demonstrate that spline curves designed using discrete conformal mapping exhibit superior smoothness and reduced distortion compared to centroid mapping, effectively meeting the design requirements for RPD frameworks. Mean value of interior angles between adjacent polyline segments is utilized as a quantitative metric for evaluating the smoothness of the polyline, where smaller angles correspond tosuperior smoothness. The mean value can be calculate using the Equation 1.

$$L_{smoothness}=\sum_{i=0}^{n-2}\arccos(\frac{{\vec{l}}_{i}\cdot {\vec{l}}_{i+1}}{\left\|{\vec{l}}_{i}\right\|.\left\|{\vec{l}}_{i+1}\right\|})/(n-1)$$ (1)

where ${\vec{l}}_{0},{\vec{l}}_{1}...{\vec{l}}_{n-1}$ are the vector representation of each polyline segment that constitutes the polyline *L*. These segments are connected end to end. Computational analysis reveals mean values of 0.067 rad for Fig 3(b) and 0.121 rad for Fig 3(d).

After generating the 2D planar parameterized mesh of the dental model, spline curve editing is performed based on this parameterization. The process comprises the following steps:

① Control Point Selection and Mapping: Control points are sequentially selected on the 3D dental model to define the spline curve. These points are then projected onto the 2D parameterized mesh, enabling spline curve construction within the planar domain.

② Curve Discretization and Intersection Calculation: The constructed spline curve in the planar domain is discretized into a series of line segments. The intersections between this discretized spline polyline and the parameterized mesh are computed to ensure accurate correspondence.

③ Back Mapping to 3D: The intersection points obtained in the planar domain are mapped back onto the 3D dental model, completing the editing process and yielding the desired spline curve on the 3D surface.

The subsequent sections detail the mapping process between the 2D parameterized plane and the 3D mesh surface, as well as the spline curve editing performed on the 2D parameterized plane in step ②:

(1) Mapping between the 2D parameterized plane and the 3D mesh surface

Assume a point p lies within a triangle T, defined by three vertices $V_{0},V_{1},V_{2}$. The triangle can be described as a convex polygon enclosing p. The positional relationship between p and the vertices $V_{0},V_{1},V_{2}$ can be expressed using barycentric coordinates as follows:


$$\begin{array}{c} p=\alpha_{0}V_{0}+\alpha_{1}V_{1}+(1-\alpha_{0}-\alpha_{1})V_{2},\\ 0\leq \alpha_{0}\leq 1,0\leq \alpha_{1}\leq 1,0\leq \alpha_{0}+\alpha_{1}\leq 1 \end{array}$$ (2)

**Fig 4:**
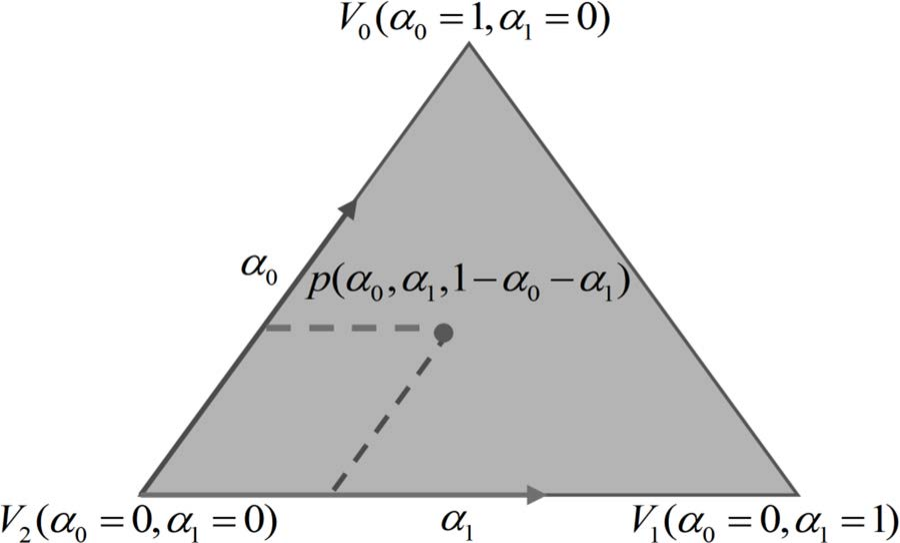
Diagram of the relative position of a point *p* within a triangleT.

By solving this system of equations, the relative positional relationships $\left(\alpha_{0},\alpha_{1},1-\alpha_{0}-\alpha_{1}\right)$ between p and the triangle vertices $V_{0},V_{1},V_{2}$ can be determined, as illustrated in Fig 4. Within any triangle, this relationship enables a mutual mapping between any point on the 2D parameterized plane and the corresponding 3D mesh surface using the Equation 2.

(2) Spline Curve Editing on the 2D Parameterized Plane

The cubic quasi-uniform B-spline curve utilized in this study serves as the foundation for the modeling process. Its control points are selected sequentially on the 3D dental model and mapped onto the 2D parameterized plane, where the spline curve is constructed and maintained as continuous.

To project the curve back onto the 3D dental model, the intersection points between the spline curve and the 2D parameterized mesh must be calculated. This involves uniformly sampling along the continuous spline curve to generate a series of sampling points. Using these points, line segments are formed between adjacent samples, and their intersections with the parameterized mesh are computed sequentially.

Once all ordered intersection points are identified in the 2D plane, they are mapped back onto the 3D mesh surface, thereby completing the spline curve editing process on the 3D dental model. This approach ensures precise correspondence between the 2D and 3D domains while maintaining the geometric integrity of the spline curve.

**Fig 5:**
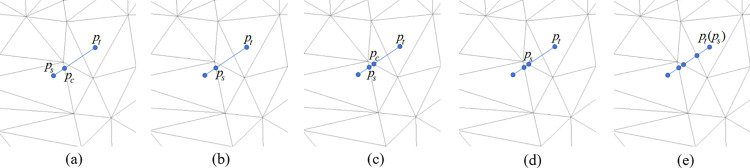
Intersection calculation process of the spline curve on the parameterized plane.

Assume that the starting point $p_{s}$ and the end point $p_{t}$ are two adjacent sampling points on spline curve in the 2D parameterized plane (Fig 5), the intersection calculation process is as follows:

① If the starting point $p_{s}$ lies within a triangular face of the mesh, calculate the intersection point $p_{c}$ where the line segment ($p_{s}$ *p*_*t*_) intersects with one of the triangle’s edges, update $p_{s}$ to the intersection point $p_{c}$, as illustrated in Fig 5(a) and 5(b).

② If the starting point $p_{s}$ lies on an edge shared by two triangles, identify which of the two triangles is intersected by the line segment ($p_{s}$ *p*_*t*_), calculate the intersection point $p_{c}$ on the corresponding edge, update $p_{s}$ to the intersection point $p_{c}$, as shown in Fig 5(c) and 5(d).

③ Repeat the above steps iteratively, updating $p_{s}$ to $p_{c}$ after each intersection, until the end point $p_{t}$ is reached, as shown in Fig 5(e).

**Fig 6:**
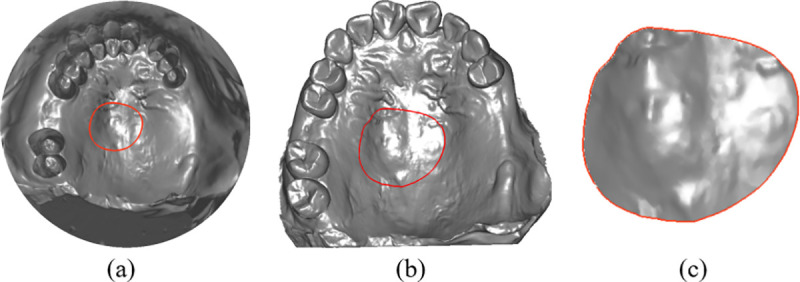
Spline curve design and trimming. **(a)** Spline curve generation on the parameterized plane. **(b)** Mapping of the spline curve from the parameterized plane to the 3D model. **(c)** Clipped mesh.

After discretizing the spline curve on the 2D parameterized plane, it is mapped back onto the 3D dental model, ensuring that the discrete spline curve is strictly constrained to the mesh surface. To achieve this, all triangular faces intersected by the spline curve on the 3D dental model are subdivided based on the mesh topology. The resulting region is then clipped, with the discrete closed spline curve defining the boundary, as shown in Fig 6.

### 3.2. Component modeling based on spline curves

**Fig 7:**
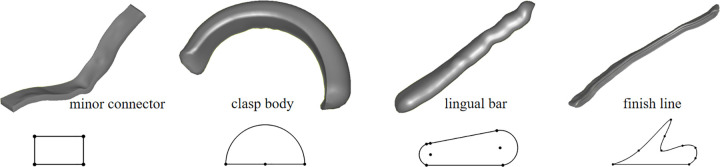
Component models based on surface spline curves and their cross-sectional shapes.

In the RPD framework design system, components such as lingual bars, minor connectors, clasps, and finish lines are generated in real-time on the 3D dental model, as shown in Fig 7. These components exhibit the following characteristics:

(1) They are strip-shaped structures that follow the trajectory of the spline curves.
(2) Each component has a defined width and a specific cross-sectional shape.
(3) The base of each component conforms closely to the surface of the 3D dental model.
(4) The components are dynamically adjusted in real-time in response to spline curve edits, ensuring immediate adaptation to design changes.

To achieve real-time modeling of component structures, this study proposes a spline curve modeling technique constrained by a 3D mesh surface. In the parameterized plane, the spline curve acts as a “guide line”, with equidistant segments constrained to the 3D dental surface along the curve. This creates a “strip-shaped” surface that conforms to the dental surface, and the final mesh model is generated by offsetting this surface. The modeling process is as follows:

(1) Spline Curve Generation

Open spline curves on the 3D dental model are generated using the spline curve editing method described in Section 3.1.

(2) Uniform Sampling

**Fig 8:**
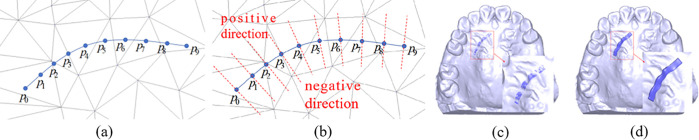
Component modeling process based on surface spline curves. **(a)** Equidistant sampling points of the spline curve in the parameterized plane. **(b)** Generating line segments based on spline curve sampling points. **(c)** Constructing a surface fitting the dental model. **(d)** Offsetting to generate the component model.

The 3D spline curve is uniformly sampled to obtain a series of ordered, equidistant sample points. These points are then mapped to the corresponding 2D spline curve, as shown in Fig 8(a).

(3) Normal Direction Sampling

On the 2D parameterized plane, at each sample point, two line segments are drawn along the normal direction to the curve (in both positive and negative directions). The lengths of these segments are equal, ensuring their sum matches the width of the component in 3D space. Uniform sampling is then performed along these line segments, and the positions of the sampling points on the parameterized plane (including the triangle and relative position) are computed, as shown in Fig 8(b).

(4) Mapping to 3D Surface:

The sampling points from the line segments in Step 3 are mapped back onto the 3D dental mesh model. These points are then used to construct a surface that conforms to the dental model, as illustrated in Fig 8(c).

(5) Component Generation

Based on the type of component being modeled, an offset distance is assigned to each vertex on the surface. A surface shelling operation is then performed to generate the component models, as shown in Fig 8(d).

In step (5), directly offsetting the constructed surface may result in mesh self-intersections, especially in regions with concavities or when the offset distance is large. These self-intersections can hinder subsequent Boolean operations. To ensure real-time modeling, self-intersection correction is not applied during the modeling process. Instead, detection and repair of self-intersections are performed prior to Boolean operations, maintaining the integrity of the final model.

## 4. Mesh surface offset

In computer-aided design and manufacturing, mesh surface offsetting is a fundamental geometric problem with significant applications in areas such as model shelling, CNC toolpath generation, and robotic path planning [26,27]. Mesh offsetting methods are generally categorized into two types: direct offsets based on geometric elements (points, lines, and surfaces) [28] and mesh reconstruction offsets based on distance fields [29,30].

(1) Direct Offsets Based on Geometric Elements

This method offsets geometric elements along a specified direction by a given distance. While simple, it often results in self-intersections when applied to complex models or large offset distances, presenting challenges for subsequent geometric operations.

(2) Mesh Reconstruction Offsets Based on Distance Fields

This approach uses implicit surface reconstruction to offset the mesh model, effectively avoiding self-intersections. However, it tends to have lower accuracy, with the offset mesh deviating significantly from the original model. Despite this limitation, it is well-suited for subsequent mesh editing tasks.

(3) Application in RPD Framework Design

**Fig 9:**
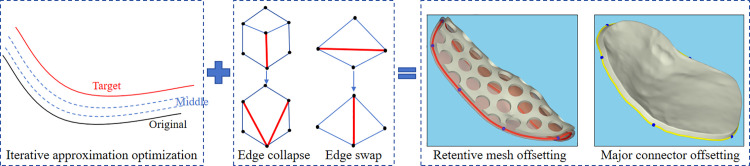
The mesh surface offset based on the iterative approximation method.

In the design of removable partial denture (RPD) frameworks, mesh offsetting is used to generate denture base connectors, major connectors, and other components. An iterative approximation method is applied to offset the mesh surface and produce these components, as shown in Fig 9. To address potential self-intersections in the offset mesh, techniques such as edge flipping, edge collapsing, and sharp vertex smoothing are integrated during the iterative offsetting process.

Let the desired offset distance be d, and set the incremental offset distance per iteration to 0.15 mm. The total number of iterations is given by n=⌈d/0.15⌉. During each iteration, the following steps are performed:

Edge Flipping: Applied to the entire mesh model to optimize mesh quality.

Edge Collapsing: Performed on edges shorter than a specified threshold, set to 0.5 times the average edge length of the mesh.

Vertex Smoothing: Applied to sharp vertices corresponding to edges with a dihedral angle exceeding 150°.

Distance Adjustment: The distance of each vertex in the offset mesh relative to the original mesh is calculated, and the position of each vertex is fine-tuned based on the desired offset distance for the iteration to ensure accuracy.

Fig 9 illustrates the application of this algorithm in generating the denture base connectors and major connectors within the RPD framework. Specifically, the denture base connector is created using two-step uniform offsets, while the major connector is generated through non-uniform offsets. After the offsetting process is complete, the original mesh and the offset mesh are stitched together to form a shell model, ensuring structural integrity and design precision.

## 5. Texture mapping modeling

**Fig 10:**
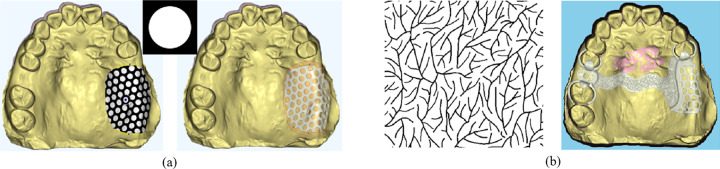
Texture mapping modeling. **(a)** Mesh hole generation on the denture base connector based on hole texture. **(b)** Wax patterns generation based on tree-like texture pattern.

In the design of removable partial denture (RPD) frameworks, it is crucial to incorporate adjustable holes on the denture base connector and branch-like patterns on the surface of the major connector, as illustrated in Fig 10.

For the denture base connector, the designer selects the appropriate hole shapes and sizes based on specific design requirements. The positions and arrangement of these holes are then adjusted to align with the shape and geometry of the components, as shown in Fig 10(a).

For the major connector, the designer chooses a suitable branch-like pattern and applies it to generate protruding structures on the mesh surface. These patterns are carefully tailored to address both functional and aesthetic considerations, as depicted in Fig 10(b).

To achieve the desired functionality, this study uses a modeling approach based on texture mapping, where hole images or branch-like texture patterns are mapped onto the mesh surface. The positions of the holes or “branches” on the 3D mesh model are identified, allowing for the direct generation of denture base connector holes and wax patterns on the mesh surface.

The quality of mesh parameterization is critical in determining the size, shape, and distribution of holes, as well as the form of the wax patterns. If the parameterization does not preserve area, the holes or patterns will be unevenly distributed. Likewise, if angles are not preserved, the hole distribution may become irregular and the pattern may suffer distortion, both of which can negatively affect the design quality.

To address these challenges, this paper employs the As Rigid As Possible (ARAP) parameterization method [31]. This method effectively balances angle and area preservation, ensuring high-quality hole and wax patterns generation.

**Fig 11:**
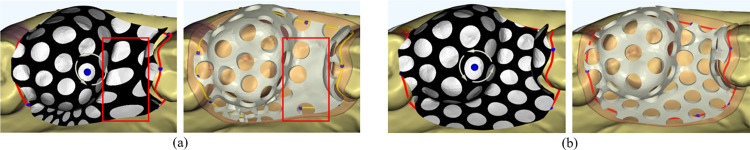
Comparison of denture base connector hole results using different parameterization methods. **(a)** LSCM parameterization mesh hole. **(b)** ARAP parameterization mesh hole.

Fig 11 shows the results of hole generation for the denture base connector using both Least Squares Conformal Maps (LSCM) [32] and ARAP parameterization. The comparison highlights that holes are more uniformly distributed with ARAP parameterization, demonstrating its advantage in maintaining design consistency and quality.

**Fig 12:**
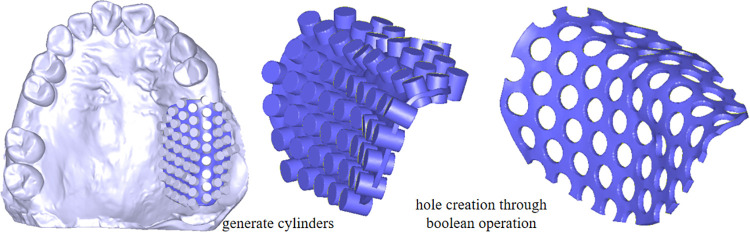
Denture base connector generation.

Using ARAP parameterization, the hole image is accurately mapped onto the denture base connector surface. The centers of the holes are determined based on the mapped texture, ensuring precise localization on the 3D mesh. At each identified hole center, a small cylindrical structure is constructed, with its top and bottom surfaces entirely positioned outside the denture base connector component. This ensures that the cylinder fully intersects with the mesh at the desired locations. As shown in Fig 12, Boolean subtraction operations are applied between the denture base connectors and the constructed cylinders, resulting in the generation of clean and precise holes on the mesh surface. This approach ensures geometric accuracy and maintains the structural integrity of the denture base connector.

The wax patterns are designed to create branch-like protrusions on the framework surface, primarily to improve phonetic function by shaping the framework to better support speech articulation. To achieve this, a predefined pattern image is mapped onto the local surface of the framework using the ARAP (As-Rigid-As-Possible) parameterization method. This mapping ensures accurate alignment of the pattern with the framework geometry.

**Fig 13:**
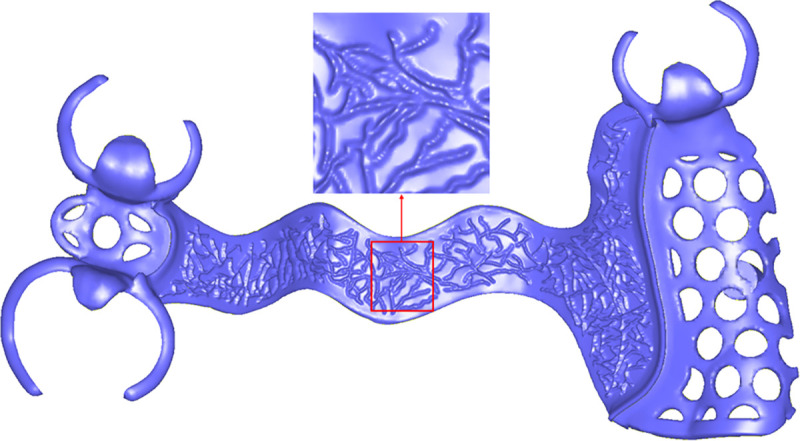
Wax patterns generation.

Based on the mapping results, the triangles within the mesh that correspond to the pattern areas are identified. These triangles are subsequently subdivided and deformed to form the branch-like protrusions. The subdivision process involves dividing the selected triangles into smaller elements, allowing for finer geometric adjustments. The deformation step ensures the protrusions align seamlessly with the underlying mesh structure, maintaining geometric consistency, as shown in Fig 13.

## 6. Component models fusion

The component models in the above design process must be merged to complete the preliminary design of the RPD framework. 3D geometric model fusion is a key technique for integrating multiple independent models into a single, unified geometry, ensuring overall consistency and functionality. The fusion techniques employed can vary depending on the specific application scenario. In the field of CAD modeling, the models to be fused are closed 3D models. To ensure high modeling accuracy, 3D Boolean operations are typically employed to achieve precise “rigid fusion” of the models [33,34]. In fields such as gaming and artistic modeling, open component models are often assembled onto open or closed “parent” models. The focus of model fusion in these areas is on the resulting form after fusion. Transition fusion techniques are typically employed to seamlessly and smoothly integrate the component models into the “parent” model [35,36].

**Fig 14:**
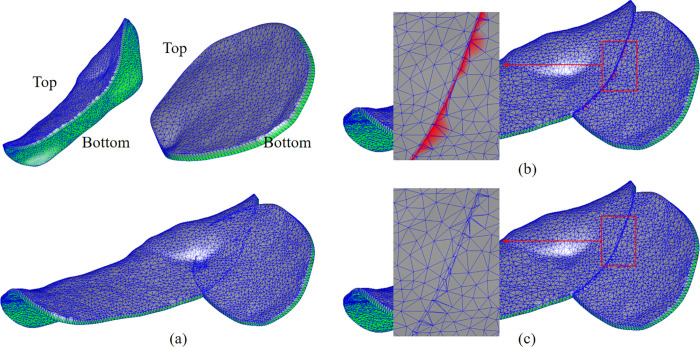
Boolean operation based on vertex attributes. **(a)** Model with “top” and “bottom” attribute labels. **(b)** Boolean operation with vertex attribute “labels”. **(c)** Re-labeling of “top” and “bottom” attributes at intersection points.

To ensure the precision and functionality of the fused models in this study, the following two criteria for 3D model fusion are proposed:

(1) The fusion process must preserve the geometric precision of each component model and ensure smooth transitions within the fused region.
(2) To facilitate interactive editing of the “top” surface of the fused model, it is essential to distinguish the “top” and “bottom” surface attributes of the fused model based on the corresponding attributes of the original component models after fusion, as illustrated in Fig 14(a).

To meet the above requirements for model fusion, this study adopts an enhanced Boolean operation approach to achieve model integration. The proposed algorithm assigns attribute labels to the vertices of the original component models prior to the fusion process. During the intersection calculations, triangle subdivision, and regularization operations, the attributes of the original component model vertices are tracked, and intersection points generated during these operations are marked. As shown in Fig 14(b), the red points represent the intersection points resulting from the Boolean operations.

Following the Boolean operations, the “top” and “bottom” attributes of each intersection point are re-labeled based on the attributes of neighboring vertices. This ensures accurate classification and proper distinction of the fused model’s surface properties, as illustrated in Fig 14(c).

**Fig 15:**
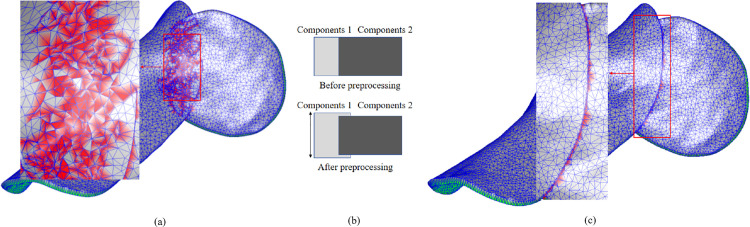
Model preprocessing improves Boolean operation results. **(a)** Boolean operation result without preprocessing. **(b)** Schematic diagram of component model preprocessing. **(c)** Boolean operation result with preprocessing.

Additionally, since the “bottom” surfaces of the component models are obtained by trimming the dental arch model, partial surface overlap occurs between the “bottom” surfaces of adjacent component models. Furthermore, when the offset distances of the two models are equal, local overlap can also occur on the “top” surfaces. These overlaps pose significant challenges to the robustness of the Boolean operations. Moreover, the mesh quality in these overlapping regions tends to degrade due to re-meshing during the intersection calculations, which adversely affects subsequent editing operations, as shown in Fig 15(a).

To address this issue, this study optimizes both the Boolean operation algorithm itself and the preprocessing of the component models prior to the Boolean operations. These improvements significantly enhance the robustness and mesh quality of the Boolean fusion process.

To improve the robustness of Boolean operations, small random perturbations are introduced to the mesh vertices during intersection calculations to prevent coplanar intersecting triangles. For preprocessing of the component models, as shown in Fig 15(b), prior to executing the Boolean operation between two models, the vertices on the “top” and “bottom” surfaces of one model are slightly displaced along their respective normal vectors. This displacement ensures that no self-intersections occur within the original model. In this study, the displacement distance is set to 0.15 mm, which is an experience-based reference value. During the Boolean operation, these displaced vertices are labeled with the “Moved” attribute. After the operation is complete, the “Moved” vertices in the fused model are shifted back by 0.15 mm, preserving the local mesh topology and maintaining consistency with the original model, as illustrated in Fig 15(c).

**Fig 16:**
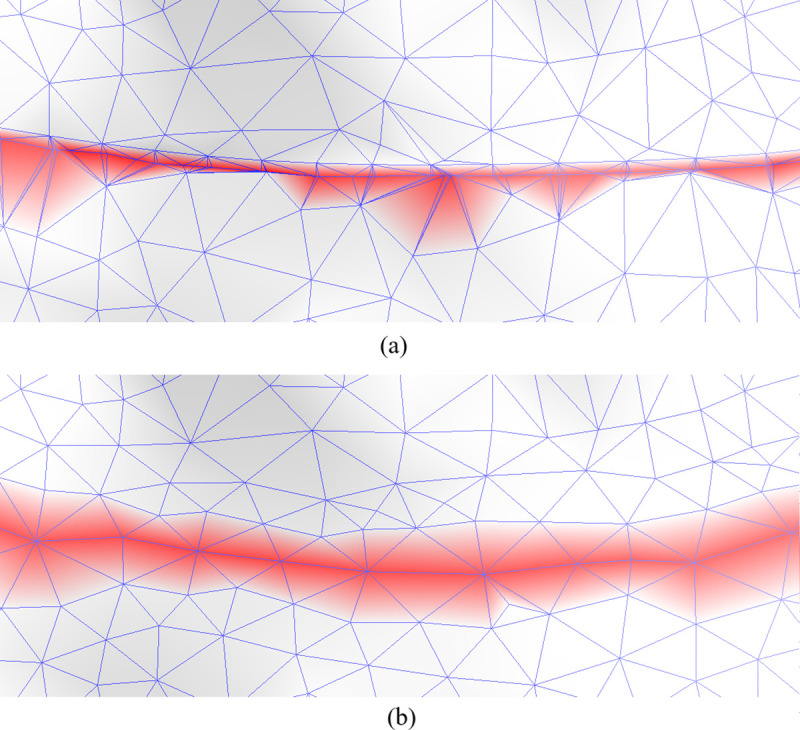
Intersection area optimization. **(a)** Before optimization. **(b)** After optimization.

Following the Boolean operation, “ridges” inevitably form on the “top” and “bottom” surfaces of the components due to the intersections. These ridges disrupt the smoothness of the surfaces and degrade mesh quality, as shown in Fig 16(a). To mitigate this, local smoothing and simplification techniques are applied to the intersection points and their neighboring vertices. This post-processing step ensures that the resulting mesh meets the desired quality standards, as illustrated in Fig 16(b).

## 7. Related function testing and clinical application

The development environment for the RPD framework design system proposed in this study is Visual Studio 2019, with C++ as the programming language and Windows 10 as the operating system. While the quality of RPD framework design is primarily determined by the designer’s expertise, design software plays a crucial role in providing technical support. The accuracy and quality of modeling provided by the software directly influence the overall design outcome. This section evaluates the superiority of the proposed design system based on the results of spline curve editing, denture base connector hole generation, and mesh offset operations.

### 7.1. The “smoothness” of spline curve editing

Spline curve editing is a critical technology for modeling various components within the software system. The algorithm proposed in this study converts 3D spline curve editing into 2D using a conformal parameterization method. This approach greatly enhances the efficiency, robustness, and local control precision of the spline curve editing process.

Once the spline curve is drawn, local modifications are typically made by adjusting a “control point” along the curve. It is essential that the local shape of the spline curve transitions smoothly during this process. Such smooth transitions reduce the editing workload and minimize the overall design time.

**Fig 17:**
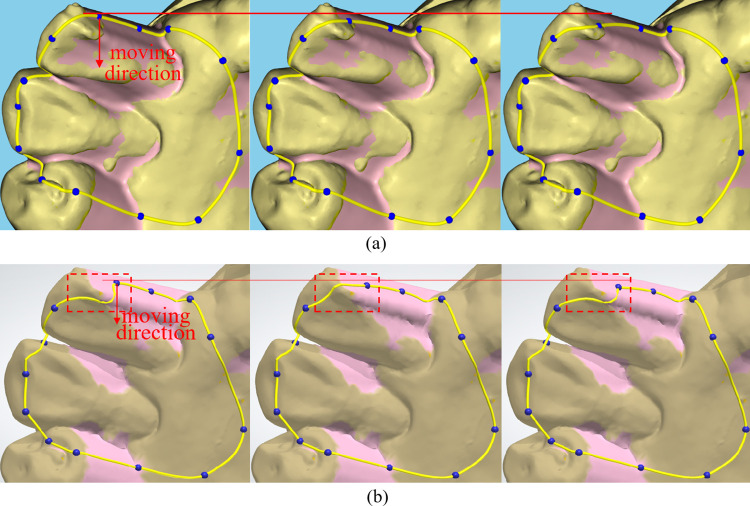
Comparison of the “smoothness” in spline curve editing. **(a)** Editing process using the algorithm in this paper. **(b)** Editing process using 3Shape software.

Fig 17 compares the algorithm proposed in this study (Fig 17(a)) with the spline curve editing process in 3Shape software (Fig 17(b)). A control point is dragged on the model surface. The proposed algorithm ensures a smooth transition in the spline curve’s shape during control point adjustments. In contrast, the 3Shape algorithm results in abrupt shape changes, making it less effective for local shape modifications during the spline drawing process.

### 7.2. Uniform distribution of denture base connector holes

The denture base connector in an RPD framework ensures denture stability, prevents dislodgement, distributes chewing forces, and protects abutment teeth, thereby helping to preserve the health of both the natural teeth and the alveolar bone. The shape and size of the holes significantly influence the strength of the denture base connector.

**Fig 18:**
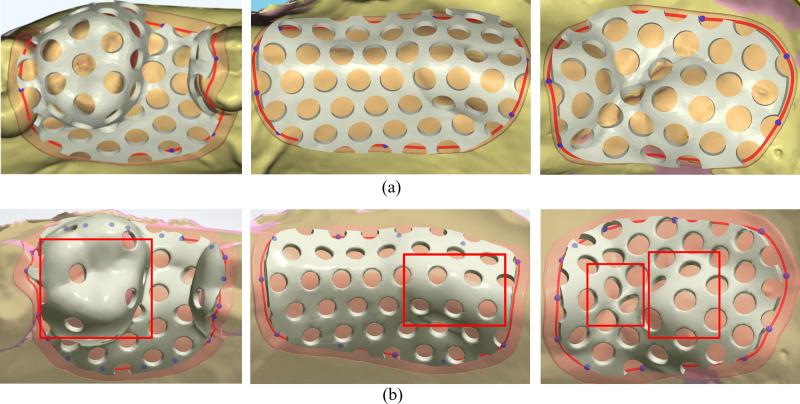
Comparison of the uniformity of three sets of denture base connector hole distributions. **(a)** Results from the proposed algorithm. **(b)** Results from the 3Shape algorithm.

To evaluate the reliability of the algorithm proposed in this study, a comparison was conducted with the denture base connector hole design implemented in the 3Shape software. In this comparison, denture base connectors were generated at the same locations on different dental models to assess the uniformity of the hole distribution. Fig 18(a) presents three sets of denture base connectors generated using the proposed algorithm, while Fig 18(b) shows the denture base connectors created by the 3Shape software at the corresponding positions on the same models. The first set of meshes was tested on a high-curvature surface under extreme conditions, while the second and third sets were tested under normal conditions. The results clearly demonstrate that the proposed algorithm achieves superior hole distribution uniformity, especially on high-curvature regions.

### 7.3. Accuracy and quality of the mesh offset algorithm

Mesh offset is a critical technique for generating components, such as large connectors, within the software system. The accuracy and quality of the mesh offset directly impact the modeling quality of these components. To evaluate the effectiveness of the offset algorithm proposed in this study, a comparison was conducted with the mesh offset algorithm implemented in Geomagic software.

Most RPD framework components have a thickness of approximately 1.0 mm [37,38]. Table 1 presents a comparison of the performance between the mesh offset algorithm proposed in this study and the algorithm used in Geomagic software. The comparison includes the average offset error for different offset distances (ranging from 0.1 mm to 0.7 mm) and the occurrence of mesh self-intersections. As shown in the table, the proposed offset algorithm produces higher-fidelity models, effectively minimizing mesh self-intersections and improving offset accuracy across a range of distances.

**Table 1:** Comparison of mesh offset algorithms.

Test Methods	Test Content	Offset Distance						
0.1mm	0.2mm	0.3mm	0.4mm	0.5mm	0.6mm	0.7mm
Algorithm of This Paper	Average Offset Error(mm)	0.0018	0.0021	0.0022	0.0024	0.0028	0.0033	0.0034
	self-intersection	NO	NO	NO	NO	NO	NO	NO
Geomagic	Average Offset Error(mm)	0.0024	0.0035	0.0045	0.0051	0.0056	0.0062	0.0068
	self-intersection	NO	NO	NO	YES	YES	YES	YES

### 7.4. Clinical application

**Fig 19:**
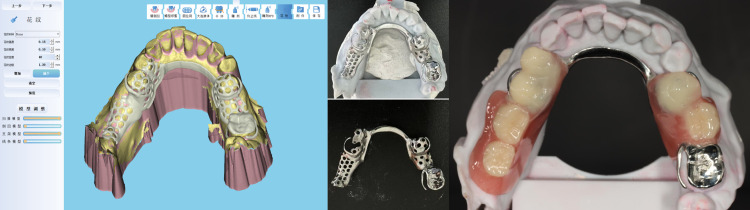
Digital design software for restorations and design cases.

The software system described in this paper has been successfully deployed in clinical practice, as shown in Fig. 19, and has been used to complete more than 30,000 clinical designs.

## 8. Summary

This paper presents a comprehensive computer-aided design (CAD) system for removable partial denture (RPD) frameworks. The system enables a fully digital workflow, from digitized dental model input and framework design to the generation of final models ready for direct 3D printing. It is built around several key technologies, including spline curve editing, high-precision mesh offsetting, enhanced Boolean-based component fusion, and texture-based modeling using ARAP parameterization, and has been validated in over 30,000 clinical cases.

The main contributions of this work are as follows:

(1) Complete RPD framework CAD system: The system supports the design of all major components, including denture base connectors, major and minor connectors, clasps, rests, wax patterns, and finish lines, providing an engineering- and commercial-grade solution for clinical application.
(2) Spline curve editing using conformal mapping: Discrete conformal mapping is employed to parameterize the dental model onto a planar disk, enabling high-smoothness, low-distortion curve editing with real-time interactivity. Both closed and open curves are supported for constructing various framework components.
(3) High-precision, self-intersection-free mesh offsetting algorithm: Iterative approximation combined with adaptive mesh simplification maintains offset accuracy while avoiding self-intersections, suitable for generating shells of critical structures such as denture base and major connectors.
(4) Enhanced Boolean operations for component fusion: Multiple components are accurately and seamlessly integrated, with intersection regions smoothed and simplified. Top and bottom surface attributes are preserved, resulting in framework models that balance structural accuracy and aesthetics.
(5) Texture-based modeling using ARAP parameterization: Precise generation of denture base connector holes and branch-like wax patterns on major connectors is achieved, ensuring uniform hole distribution and shape preservation, thereby enhancing both functionality and aesthetics.

The system workflow covers dental model preprocessing, generation of denture base and major connectors, modeling of minor connectors and clasps, Boolean-based component fusion, and local wax pattern generation. It is supplemented with auxiliary tools, including thickness analysis and interference detection, to enhance design experience and stability.

In summary, the proposed CAD system introduces innovations in digital geometry processing and provides a practical, commercial-grade solution, offering essential technical support for the digital design of RPD frameworks.

## 9. Limitations and future work

Although the system has demonstrated high efficiency and reliability in designing removable partial denture (RPD) frameworks, the inherently personalized nature of RPDs means that the current design workflow still relies heavily on the technician’s experience. This reliance can result in variability in design quality among different technicians.

Future work will focus on developing intelligent RPD framework design using artificial intelligence techniques. This includes the systematic collection of key patient data, intelligent evaluation of design solutions, and automated generation of patient-specific frameworks. Essential data may include patient demographics (e.g., age, gender), occlusal force distribution, dental arch and jaw morphology, and clinical feedback, which can inform design optimization. Design evaluation will consider metrics such as occlusal load distribution, patient comfort, long-term stability, and aesthetic quality to ensure high-quality and reproducible outcomes.

Through these advancements, RPD CAD systems are expected to evolve from experience-driven platforms to data-driven and intelligent design systems, facilitating efficient, accurate, and patient-specific denture framework design, and further advancing the clinical and engineering applications of digital prosthodontics.

## Supporting information

S1 Video Complete design process video. (MP4)
